# Phenotypic Variation in Vietnamese Osteogenesis Imperfecta Patients Sharing a Recessive *P3H1* Pathogenic Variant

**DOI:** 10.3390/genes13030407

**Published:** 2022-02-24

**Authors:** Lidiia Zhytnik, Binh Ho Duy, Marelise Eekhoff, Lisanne Wisse, Gerard Pals, Ene Reimann, Sulev Kõks, Aare Märtson, Alessandra Maugeri, Katre Maasalu, Dimitra Micha

**Affiliations:** 1 Department of Human Genetics, Amsterdam Movement Sciences, Amsterdam UMC, De Boelelaan 1117, 1081 HV Amsterdam, The Netherlands; l.zhytnik@amsterdamumc.nl (L.Z.); l.wisse@amsterdamumc.nl (L.W.); g.pals@amsterdamumc.nl (G.P.); a.maugeri@amsterdamumc.nl (A.M.); 2 Department of Traumatology and Orthopaedics, Institute of Clinical Medicine, The University of Tartu, Puusepa 8, 50410 Tartu, Estonia; aare.martson@ut.ee (A.M.); katre.maasalu@ut.ee (K.M.); 3 Faculty of Nursing, College of Medicine and Pharmacy, Hue University, 06 Ngo Quyen, Hue 530000, Vietnam; binhthuybi@yahoo.com; 4 Amsterdam Bone Centre, Department of Internal Medicine Section Endocrinology, Amsterdam UMC, De Boelelaan 1117, 1081 HV Amsterdam, The Netherlands; emw.eekhoff@amsterdamumc.nl; 5 Institute of Genomics, The University of Tartu, Riia 23b, 51010 Tartu, Estonia; ene.reimann@ut.ee; 6 QEII Medical Centre, Perron, Institute for Neurological and Translational Science, 8 Verdun Street, Nedlands, WA 6009, Australia; sulev.koks@perron.uwa.edu.au; 7 Centre for Molecular Medicine and Innovative Therapeutics, Murdoch University, 90 South Street, Murdoch, Perth, WA 6150, Australia; 8 Clinic of Traumatology and Orthopaedics, Tartu University Hospital, Puusepa 8, 50410 Tartu, Estonia

**Keywords:** recessive osteogenesis imperfecta, rare disorders, bone dysplasia, rare skeletal disease, genotype-phenotype correlation, P3H1, next-generation sequencing

## Abstract

Osteogenesis imperfecta (OI) is a syndromic disorder of bone fragility with high variation in its clinical presentation. Equally variable is molecular aetiology; recessive forms are caused by approximately 20 different genes, many of which are directly implicated in collagen type I biosynthesis. Biallelic variants in prolyl 3-hydroxylase 1 (*P3H1*) are known to cause severe OI by affecting the competence of the prolyl 3-hydroxylation—cartilage associated protein—peptidyl-prolyl cis-trans isomerase B (P3H1-CRTAP-CyPB) complex, which acts on the Pro986 residue of collagen type I α 1 (COL1A1) and Pro707 collagen type I α 2 (COL1A2) chains. The investigation of an OI cohort of 146 patients in Vietnam identified 14 families with *P3H1* variants. The c.1170+5G>C variant was found to be very prevalent (12/14) and accounted for 10.3% of the Vietnamese OI cohort. New *P3H1* variants were also identified in this population. Interestingly, the c.1170+5G>C variants were found in families with the severe clinical Sillence types 2 and 3 but also the milder types 1 and 4. This is the first time that OI type 1 is reported in patients with *P3H1* variants expanding the clinical spectrum. Patients with a homozygous c.1170+5G>C variant shared severe progressively deforming OI type 3: bowed long bones, deformities of ribcage, long phalanges and hands, bluish sclera, brachycephaly, and early intrauterine fractures. Although it remains unclear if the c.1170+5G>C variant constitutes a founder mutation in the Vietnamese population, its prevalence makes it valuable for the molecular diagnosis of OI in patients of the Kinh ethnicity. Our study provides insight into the clinical and genetic variation of *P3H1*-related OI in the Vietnamese population.

## 1. Introduction

Osteogenesis imperfecta (OI) is a connective tissue disorder with bone fragility as the predominant feature. The disease shows a continuum of clinical severity, the heterogeneity of which is reflected by the five clinical Sillence classification types [1,2]. Type 1 (OMIM#: 166200) is the mild end of the spectrum and includes patients who can experience a variable number of bone fractures with very minimal skeletal dysplasia [3]. Type 2 (OMIM#: 166210) is the most severe form of OI, with patients succumbing to perinatal death due to severe skeletal dysplasia [3]. Type 3 (OMIM#: 259420) is the most severe form of adult OI with bone fragility accompanied by progressive skeletal malformations. Type 4 (OMIM#: 166220) usually has a moderate disease presentation, whereas type 5 (OMIM#: 610967) is uniquely characterised by hyperplastic callus formation. Secondary features of the disease include blue sclera, deafness, dentinogenesis imperfecta (DI), as well as cardiovascular problems and pulmonary distress [3]. Despite the high prevalence and severity of the secondary OI features, no classification system exists, reflecting the inadequate comprehension of their underlying pathology. To address this, it is necessary to conduct extensive genotype-phenotype analyses.

OI is known to have a frequency of approximately 1:15–20,000, and it is determined that the vast majority of patient cases (~90%) are caused by autosomal dominant pathogenic variants in the collagen type I α 1 chain (*COL1A1*, OMIM#: 120150) and collagen type I α 2 chain (*COL1A2*, OMIM#: 120160) producing collagen type I [3]. Defects in collagen type I can also be caused by several genes affecting its regulation [4]; the prevalence of OI forms by recessive genes may be increased in isolated consanguineous populations [5]. Pathogenic variants in the prolyl 3-hydroxylase 1 (*P3H1*, OMIM#: 610339) gene were discovered in 2006 as a cause of severe recessive OI, genetic type VIII (OMIM#: 610915) [3,6]; although the clinical types 2 and 3 OI are commonly reported, milder cases also exist [7]. *P3H1* codes for P3H1, which forms a heterotrimeric complex with the cartilage-associated protein (CRTAP) and cyclophilin B (PPIB); pathogenic variants in both of the latter are also associated with OI [8,9]. P3H1 provides the enzymatic activity of the prolyl 3-hydroxylase 1 complex, which is responsible for the posttranslational 3-hydroxylation of distinct proline residues in the COL1A1 and COL1A2 chains, which are the Pro986 and Pro707, respectively. Although the role of the resulting hydroxylated 3-hydroxyproline is not clear, its decrease generally correlates with collagen over-modification, which points to delayed collagen folding [6].

A total of 71 *P3H1* OI-causing pathogenic variants have been identified [10,11,12,13,14,15,16]. Specific *P3H1* pathogenic variants are known to be prevalent in distinct populations; these include the c.1080+1G>T variant, which is common in West Africa, where it originated from tribes in Ghana and Nigeria [17,18]. The c.232delC pathogenic variant is found in the isolated Irish Traveler population where increased carrier frequency exists due to a high degree of consanguinity [19]. The c.1170+5G>C pathogenic variant has been reported in four OI patients of Vietnamese descent [16,17]. In our study, we identified the c.1170+5G>C pathogenic variant as highly prevalent (10.3%) in an OI cohort of 146 patients (121 families) in Vietnam [20]. Furthermore, we provide, for the first time, a detailed overview of the clinical characteristics and phenotypic variation of these patients, which also includes cases of milder OI.

## 2. Materials and Methods

### 2.1. Patients

Data from the study participants were extracted from the Osteogenesis Imperfecta database of the Clinic of Traumatology and Orthopedics, University of Tartu, Estonia (UT OI database). The UT OI database includes clinical, phenotypic, and genealogical information collected from OI patients and their healthy relatives during interviews [20]. During the interviews with the medical and research team of the University of Tartu in Vietnam, clinical examination and phenotype descriptions were performed, and patients were classified in concordance with the updated clinical Sillence classification (types 1–5).

### 2.2. Genomic DNA Extraction and Sanger Sequencing of COL1A1 and COL1A2

**Figure 1:**
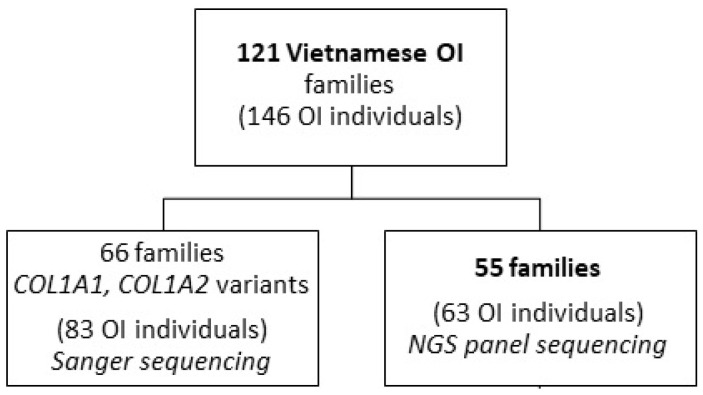
Flowchart of the Sanger sequencing and next-generation sequencing (NGS) analysis of the Osteogenesis Imperfecta (OI) Vietnamese families.

Genomic DNA extraction from EDTA-preserved blood and further Sanger sequencing and mutational analysis of the *COL1A1*, *COL1A2*, and interferon induced transmembrane protein 5 (*IFITM5*, OMIM#: 614757) genes were performed at the University of Tartu, as described previously [3,21,22]. Out of 121 Vietnamese OI families (146 OI individuals) in the database, 55 families (63 OI individuals), in which *COL1A1* and *COL1A2* causative variants were not identified, were referred for next-generation sequencing (NGS) with the OI panel (Figure 1).

### 2.3. Targeted Next-Generation Sequencing

Families negative for *COL1A1* and *COL1A2* pathogenic variants were further referred for targeted NGS using the OI panel of 41 genes at the Genome Diagnostic Laboratory of Amsterdam UMC. DNA was analysed by targeted NGS for the common OI-relating genes, which included *COL1A1*, *COL1A2*, alkaline phosphatase (*ALPL*, OMIM#: 171760), bone morphogenetic protein 1 (*BMP1*, OMIM#: 112264), cAMP response element-binding protein 3-like 1 (*CREB3L1*, OMIM#: 616215), *CRTAP* (OMIM#: 605497), FK506-binding protein 10 (*FKBP10*, OMIM#: 607063), *IFITM5*, *P3H1*, low density lipoprotein receptor-related protein 5 (*LRP5*, OMIM#: 603506), procollagen-lysine, 2-oxoglutarate 5-dioxygenase 2 (*PLOD2*, OMIM#: 601865), plastin 3 (*PLS3*, OMIM#: 300131), *PPIB* (OMIM#: 123841), serpin peptidase inhibitor, clade f, member 1 (*SERPINF1*, OMIM#: 172860), serpin peptidase inhibitor, clade h, member 1 (*SERPINH1*, OMIM#: 600943), transcription factor sp7 (*SP7*, OMIM#: 606633), transmembrane anterior posterior transformation 1 (*TAPT1*, OMIM#: 612758), transmembrane protein 38B (*TMEM38B*, OMIM#: 611236) and wingless-type MMTV integration site family, member 1 (*WNT1* OMIM#: 164820) [3]. The full list of genes analysed by targeted NGS is presented in Table S1. The analysis was performed using the Illumina sequencing platform by using the Roche NimbleGen SeqCap EZ Human Exome Library v2.0 custom-made enrichment kit (Roche, Switzerland) on an Illumina HiSeq2000 sequencer (Illumina, CA, USA). NGS data was processed with an in-house bioinformatics analysis pipeline consisting of the Burrows-Wheeler Aligner for alignment of reads, the Genome Analysis Tool Kit Lite for bam file processing [23], and Cartagenia software (Agilent Technologies, CA, USA). Variant pathogenicity was evaluated with the Alamut Visual v2.15 program (SOPHiA Genetics, Switzerland). The presence of the identified variants in the parents and siblings was examined with Sanger sequencing using primers with M13 tails. Sanger sequencing was also used to investigate the presence of *P3H1* single nucleotide polymorphisms (SNPs) in the patients. Unique primer sequences excluding M13 tails are presented in Table S2. The PowerPlex 16^®^ System (Promega, WI, USA) based on multiplex short tandem repeats (STR) DNA typing was used to confirm paternity in family VN10.

## 3. Results

### 3.1. General Description of the Cohort

A total number of 17 patients from 14 Vietnamese families harboured pathogenic variants in the *P3H1* gene, which comprises 11.6% of the whole Vietnamese OI cohort of the UT biobank (*n* = 146) [18]. The cohort included 13 pediatric and four adult patients. Ages ranged from four months to 35 years, with a mean age of 13.7 ± 10.7 years, locktime 2016 (Table 1). Eleven subjects (64.7%) were female, and six (35.3%) were male.

**Figure 2:**
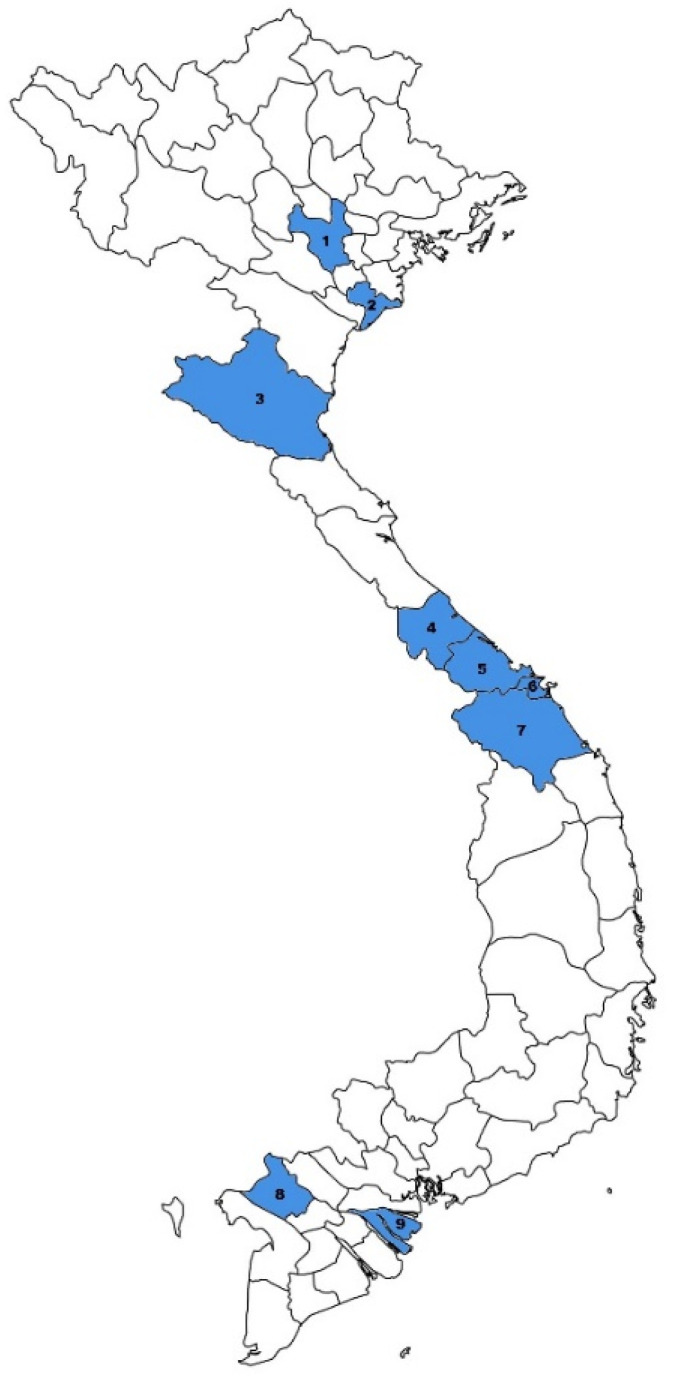
Vietnam provinces of patients’ residence. 1—Hanoi; 2—Nam Dinh; 3—Nghe An; 4—Quang Tri; 5—Thua Thien-Hue; 6—Da Nang; 7—Quang Nam; 8—An Giang; 9—Ben Tre.

**Figure 3:**
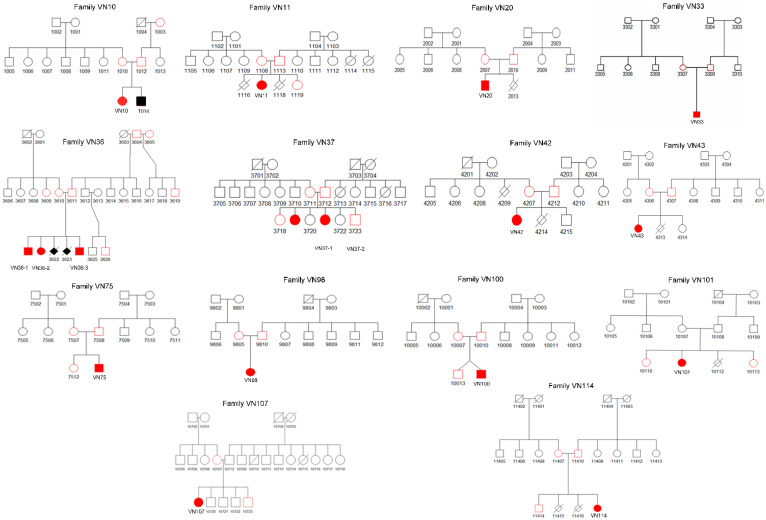
Patient pedigree trees. Squares indicate males, circles indicate females, diamonds indicate individuals of unknown sex. Affected individuals are pictured with filled-in symbols. Deceased family members are represented with a cross line. Patients and family members whose samples were available for the study are indicated in red.

Patients originated from different provinces of Vietnam, but all families belonged to the Kinh ethnic group (Figure 2). None of the families had history of consanguinity or history of OI in previous generations. In seven families, parents reported previous miscarriages. However, only in family VN36 was it clinically confirmed that the two miscarriages were prenatally fatal (Figure 3).

Patients in three families (VN11, VN20, and VN36-1, VN36-2, VN36-3) received bisphosphonate treatment prior to the phenotype descriptions in this study.

### 3.2. Genetic Analysis

The index patient of the 55 OI families was analysed by a targeted NGS panel for the common OI-related genes and revealed 17 patients in 14 families with a *P3H1* (NM_022356.3) pathogenic variant (Table 2). The c.1170+5G>C p.? pathogenic variant was identified in 15 patients of 12 families; in 13 patients, it was found in homozygous state and in two patients, VN10 and VN42, it was compound heterozygous with the c.2197A>G p.(Lys733Glu) and c.1934G>A p.(Cys645Tyr) variants, respectively. Furthermore, patient VN101 was found to have the homozygous c.1224-79G>A variant, whereas patient VN75 had this variant in compound heterozygous form with c.257del p.(Glu86Glyfs*22). All identified variants apart from c.1170+5G>C were absent from the OI variant database. All pathogenic variants were confirmed by Sanger sequencing to be present in the patient parents whose gDNA sample was available (Table 2). The only exception was one of the parents in families VN10 (Table 2). Paternity was confirmed in family VN10, which raises the possibility that the c.2197A>G variant is de novo. However, we cannot exclude the possibility of gonadal mosaicism in the father or monoallelic state of the *P3H1* variants.

Among the unaffected siblings, we found three heterozygous carriers of the c.1170+5G>C (families VN11, VN37, VN107) and one carrier of the c.1124-79G>A (family VN101) variant. Other unaffected siblings tested negative for the pathogenic variant found in the index patient of the family (Figure 3). In order to explore the genetic variation in the *P3H1* gene and possible common haplotype between carriers of the same c.1170+5G>C variant, we examined the presence of the c.465+489delG and c.465+499delG SNPs in the 14 index patients. All index patients contained the two SNPs in the homozygous state, with the exception of patient VN101, in whom they were found in the heterozygous state.

### 3.3. Clinical Characteristics

In accordance with the updated clinical OI Sillence classification, the patients were assessed to have developed the following OI types: 1 (5.9%, *n* = 1), 3 (64.7%, *n* = 11), and 4 (29.4%, *n* = 5).

The mean total number of fractures per patient was 17.9 ± 11.7 (0–40), whereas the median number of fractures per year was 1.4 (0–8). Patient VN10, who was 13 y.o., did not have any fractures; however, she was suspected of having OI, based on light-blue eye sclera, DI, and brother with clinically diagnosed OI. She and her affected brother (not included in the analysis due to unavailability of gDNA sample) were the only cohort patients able to ambulate without assistance. The mean number of fractures per year was 3.3 times higher in pediatric patients (*n* = 2.2) compared to adults (*n* = 0.7). Only ten patients (58.8%) experienced intrauterine fractures.

**Figure 4:**
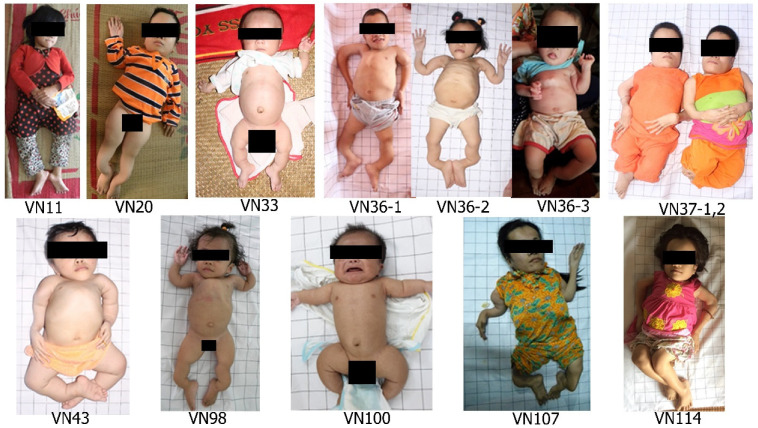
Patients with homozygous c.1170+5G>C *P3H1* pathogenic variants. Patients share typical limb, chest, and spine deformities and disproportionally long phalanges.

**Figure 5:**
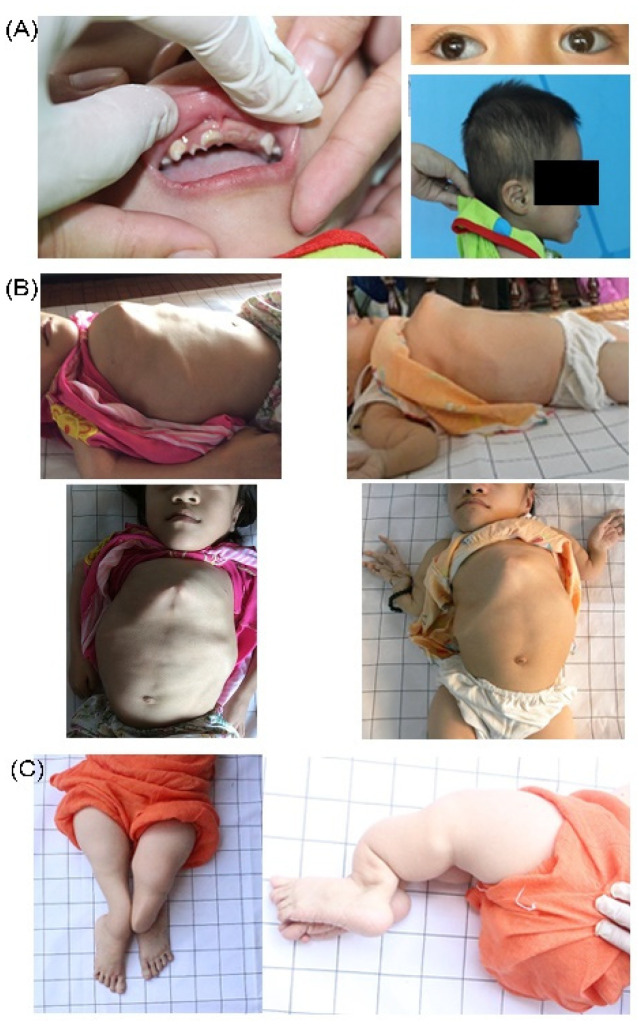
Clinical features of patients with *P3H1* pathogenic variants. (**A**) Oral features, bluish eye sclera, craniosynostosis and low-set ears; (**B**) Pectus carinatum; (**C**) Lower limb deformities.

Newborn patients VN33 and VN100 (up to 1 y.o.) were male and shared bowed legs (“frog-leg posture”) and hydrocele (Figure 4). Patients showed signs of rhizomelia, with shorter and more severely affected and deformed lower limbs. Limb bowing was noticed directly after birth. Individuals had long hands in comparison to forearms and disproportionally long phalanges. Thirteen patients (76.5%) developed chest deformities, and four patients (23.5%) had extremely severe pectus carinatum (VN36-3, VN75, VN101, VN114). Severe kyphoscoliosis was also typical among this cohort (Figure 4 and Figure 5).

The majority of patients (14/17, 82.4%) developed brachycephaly. Out of them, ten (58.9%) had typical OI triangular faces, while others had long rounded faces. Patients also showed a tall forehead with frontal bossing and low set ears. Patient VN33 had a soft unmineralised skull (Figure 4 and Figure 5).

Only three patients (VN98, VN101, and VN107) had white sclera. Blue (i.e., blue, grayish, light-blue) sclerae was common in the majority of patients (*n* = 14, 82.4%). Almost half of the cohort (*n* = 8, 47.1%) suffered from DI, and only two individuals had hearing loss (VN11, age 10 and VN107, age 33).

### 3.4. Radiographic Features

**Figure 6:**
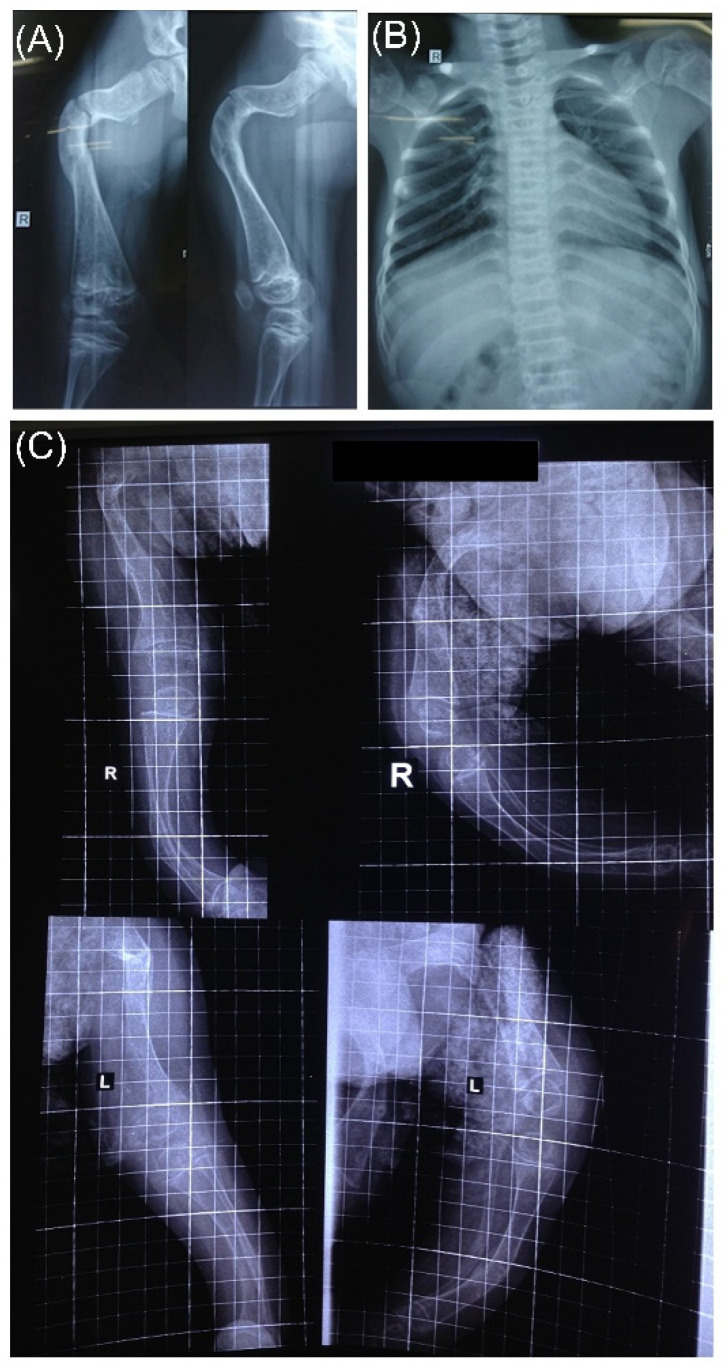
Radiographs of patients VN11 and VN75. (**A**) Radiograph of right lower limb of patient VN11; (**B**) chest radiograph of patient VN11; (**C**) radiograph of lower limbs of patients VN75, R, indicates right; L, indicates left lower limbs.

Based on available radiographs of patient VN11, both the cortical and trabecular bone mass of the proband are similarly decreased (Figure 6A,B). Femural shift is observed, and the knee joint is abnormal and deformed. Lower limbs reveal bowing, widening, and shortening of diaphyses. Popcorn-shaped abnormalities in the metaphyses (i.e., bulbous metaphyses) can be noted. Thin ribs reveal decreased bone mass.

Similarly, lower limbs’ diaphyses bowing, shortening, and widening together with bulbous metaphyses were observed in radiographs of patient VN75 (Figure 6C). Furthermore, here the cortical and trabecular bone mass were decreased.

## 4. Discussion

Despite harbouring variants in the same *P3H1* gene, the phenotype of patients varied extensively in terms of severity and clinical OI features and ranged from type 1 to type 3, 4, and even 2, reported as two confirmed OI miscarriages in family VN36. This data underlines the broad range of possible phenotype variability in cases of *P3H1*-associated OI, which lacked previous attention.

Previous studies have confirmed severe presentation of OI as a result of biallelic *P3H1* pathogenic variants. Severe adult type 3 and perinatal lethal type 2 OI have been described in numerous cases as a result of loss of function defects arising from mRNA instability due to nonsense, frameshift, or splice site pathogenic variants [6,19,24,25,26]. A study also described the loss of the Lys-Asp-Glu-Leu (KDEL) endoplasmic reticulum (ER) retrieval sequence as a consequence of a *P3H1* pathogenic variant, which also led to a lack of intracellular P3H1 and type 3 OI [27]. It remains unclear what is the underlying reason that triggers the progression of the disease to either of the two severe types in loss of function *P3H1* defects. Considering that the expression of P3H1 and CRTAP is dependent on each other, it could be speculated that the level of CRTAP expression remains sufficient in cases of missense *P3H1* pathogenic variants, which influences the phenotypic outcome [28].

Moderate type 4 OI has been reported in cases of missense *P3H1* variants; numerous cases were documented in Turkish and other OI cohorts [7,16]. Although it is tempting to assume that maintenance of protein expression can have a protective role in diminishing disease severity, it is important to note that missense *P3H1* pathogenic variants have also been reported as a cause of type 3 OI [29,30]. However, the fact that missense pathogenic variants have not been found in type 2 OI may indeed support the presentation of a milder phenotype when protein expression is not abolished. Sustained protein expression in patients VN42 and VN10 with missense pathogenic variants may partially account for the presentation of the milder OI types 4 and 1, respectively.

It might be speculated that the OI type 1 phenotype in patient VN10, who harbours a compound heterozygous combination of a loss of function and missense variant, might be associated with a decreased amount of protein with partial functionality. It remains to be seen if the discovery of more missense pathogenic variants will further uncover more about the milder end of the *P3H1* clinical spectrum. Additionally, the severity of *P3H1*-related OI might also be influenced by modifying factors and epigenetics, as it is in the case of other OI forms [4,31,32,33,34].

Another factor that may be influencing, to a great extent, the severity of *P3H1*-related OI development is the remaining functional competence of the P3H1-CRTAP-CyPB complex in different *P3H1* defects. This has been measured by the 3-hydroxylation of COL1A1 Pro986 of patient fibroblasts on which this complex exerts its function; this is found to be highly decreased and correlates with altered electrophoretic migration of the collagen chains [6,8,19,25] (Table S3). Although the mechanistic details of the relation between the two processes are not known, this results in collagen over-modification because of the excessive exposure of collagen chains to lysyl hydroxylases and prolyl 4-hydroxylases during the delayed folding of the triple helix. As such, the molecular mechanism resembles the over-modification process observed in autosomal dominant forms of OI in which defects such as glycine substitutions in *COL1A1* or *COL1A2* lead to the same effect, albeit with normal levels of Pro986 3-hydroxylation [8]. Interestingly, *P3H1* OI cases are also found to present collagen type 5 over-modification, although this was also detectable in an unaffected member of a family with severe lethal OI [24,35,36]. However, exceptions to this correlation exist; 3-hydroxylated COL1A1 Pro986 was found to be only slightly decreased in patient fibroblasts in which the KDEL ER retention motif was lost; yet, collagen type I over-modification was detected. In this case, the remaining COL1A1 Pro986 3-hydroxylation may be accounted for by some residual activity of the KDEL-lacking P3H1 protein [27]. In all these studies, severe OI types 3 and 2 were reported, which is also commonly found in cases of COL1A1 and COL1A2 glycine substitutions [6,19,25,36]. It is noteworthy that type 3 was also found in the OI patient with moderately decreased Pro986 3-hydroxylation, which points to the contribution of additional elements in OI progression and the lack of direct causative effects by Pro986 3-hydroxylation. Unfortunately, no biochemical analyses of collagen have been performed in cases of missense pathogenic variants or milder phenotypes which would enable us to compare the state of collagen Pro986 3-hydroxylation and over-modification with the more severe cases; this is also a limitation that applies to the characterisation of our OI cohort. Another decisive parameter in the OI phenotype can be differences in the Pro986 3-hydroxylation between fibroblasts and bone cells. A patient with a *P3H1* splice pathogenic variant was reported to have 4% of Pro986 3-hydroxylation in collagen produced by fibroblasts, but this was found to be increased to 55% in bone cells [19]. Clearly, more studies are required to explain the significance of this intriguing tissue-specific finding.

Regarding the group of 13 patients with the prevalent homozygous c.1170+5G>C pathogenic variants, two patients presented the moderate type 4 OI instead of the severe type 3. Interestingly, these two patients (VN11, VN36-3) received bisphosphonate treatment, although details are lacking about the treatment type, regime, and responsiveness of the patients. Thus, it is not clear if the treatment can account for the milder phenotype compared to patients VN20, VN36-1, and VN36-2 who also received bisphosphonates, but presented nonetheless OI type 3. A reported case of moderate pediatric *P3H1*-associated OI was described to show improvement in bone quality after pamidronate therapy [7]. Pamidronate treatment may also account for the extended survival of a pediatric OI patient with the Irish traveller homozygous *P3H1* c.232delC pathogenic variant compared to lethal OI noted in other patients with the same pathogenic variant [19]. Similarly, pamidronate treatment also improved bone density in two pediatric OI type 3 patients with the homozygous splice site pathogenic variant c.2055+18G>A and the compound heterozygous c.484delG and c.2155dupC pathogenic variants [25].

We have identified four novel, previously unreported variants in our cohort. The *P3H1* 1224-79G>A variant is an intronic variant, which, according to results of in silico analysis, activates a cryptic splice donor site at the position c.1224-83. Use of this cryptic site might cause insertion of 92 nucleotides, with frameshift and presence of in-frame stop codons. Therefore, this variant is likely to cause nonsense mediated decay and haploinsufficiency of P3H1 protein leading to OI. The haploinsufficiency is also associated with the c.257del nonsense variant. The c.1934G>A, p.(Cys645Tyr) is a missense variant with a highly deleterious effect, whereas the 2197A>G, p.(Lys733Glu) variant alters the conserved C-terminal KDEL motif of P3H1, necessary for ER retention of the protein, similarly to OI type 3 patients, as described by Takagi et al. [27].

The c.1170+5G>C *P3H1* variant was found to dominate our OI cohort. This variant has been previously reported in a homozygous state in three infants of Vietnamese origin [16]. The in silico prediction for the variant shows loss of the splice donor site. Due to the absence of a cryptic splice donor site in the nearest sequence, it can be predicted that the variant causes either intron retention or exon skipping. According to the functional analysis performed by Baldridge et al., the variant results in a single base pair substitution at the splice donor site at the start of intron 6; this subsequently leads to the in-frame skipping of exon 6, resulting in deletion of 90 nucleotides [19] and 360–390 amino acid residues. It is unclear if exon 6 skipping results in no or decreased expression of a non-functional protein that has been reported to commonly associate with severe lethal phenotypes [6,19,24,25,26]. The level of mRNA has been found to be normal. Collagen analysis of cultured fibroblasts showed over-modification, but the level of Pro986 3-hydroxylation was not reported. Despite the fact that most of the homozygous c.1170+5G>C patients in our cohort did not die prenatally, they did show phenotypic similarities (Figure 4).

Considering the high frequency of the c.1170+5G>C variant in our cohort (10.3%), it is plausible that it may present a founder mutation in the Vietnamese population. We found that all index patients shared the c.465+489delG and c.465+499delG SNPs in *P3H1* in the homozygous state with the exception of one patient. Unfortunately, we did not have further information about the haplotype, which could enable the determination of an estimate of when the mutation originated. In addition, the patients in our cohort are equally distributed in provinces in distant geographic locations in Vietnam; but it is not known if they originate from a common location that would potentially point to common ancestry. It is worth noting that Vietnam is generally a country with high population movement; thus, isolated communities and consanguinity are not common. Furthermore, considering the OI cohort of 146 patients, it is unknown whether this cohort is reflective of the entire OI population in Vietnam. Although a 0.016% minor allele frequency (MAF) is known in the East Asian population, there is no data about the carrier frequency in Vietnam, which could allow us to estimate the frequency of this form of OI.

## 5. Conclusions

We identified the c.1170+5G>C variant as highly prevalent in our patient cohort of recessive OI cases in which *COL1A1* and *COL1A2* variants were excluded. This can be very informative for the molecular diagnosis of the disease, especially in a country where NGS is limited in diagnostic services. Although it is unclear what the exact percentage of the OI population in Vietnam is with the c.1170+5G>C variant, we expect that a considerable number of cases could be solved by specifically testing this variant. This could provide a cost-effective method for the molecular diagnosis of recessive OI with the potential to improve clinical counselling and treatment allocation.

## Figures and Tables

**Table 1:** Clinical characteristics of patients with prolyl 3-hydroxylase 1 (*P3H1*) (pathogenic) variants.

№	Patient ID	Age (y.o.)	Sex	Type	Total Fractures	Fractures per Year	Intrauterine Fractures	Chest Deformities	Brachycephaly	Blue Sclera	Hearing Loss	DI
1	VN10	13	F	1	0	0	No	No	No	Yes	No	Yes
2	VN11	10	F	4	27	2.7	No	Yes	Yes	Yes	Yes	Yes
3	VN20	10	M	3	10	1.0	No	Yes	Yes	Yes	No	Yes
4	VN33	1	M	4	8	8.0	No	No	Yes	Yes	No	No
5	VN36-1	16	M	3	15	0.9	Yes	Yes	Yes	Yes	No	No
6	VN36-2	12	F	3	14	1.2	Yes	Yes	Yes	Yes	No	Yes
7	VN36-3	4	M	4	6	1.5	Yes	Yes	Yes	Yes	No	No
8	VN37-1	35	F	3	20	0.6	Yes	Yes	Yes	Yes	No	No
9	VN37-2	31	F	3	18	0.6	Yes	Yes	Yes	Yes	No	No
10	VN42	11	F	4	25	2.3	No	No	No	Yes	No	No
11	VN43	12	F	3	40	3.3	Yes	Yes	Yes	Yes	No	Yes
12	VN75	5	M	3	14	2.8	No	Yes	Yes	Yes	No	Yes
13	VN98	5	F	3	20	4.0	Yes	Yes	Yes	No	No	No
14	VN100	0.25	M	4	2	NA	Yes	No	No	Yes	No	No
15	VN101	22	F	3	40	1.8	Yes	Yes	Yes	No	No	Yes
16	VN107	33	F	3	30	0.9	Yes	Yes	Yes	No	Yes	No
17	VN114	12	F	3	15	1.3	No	Yes	Yes	Yes	No	Yes

DI, dentinogenesis imperfecta; F, female; M, male; NA, not available; y.o., years old.

**Table 2:** Prolyl 3-hydroxylase 1 (*P3H1*, NM_022356.3) variants in Vietnamese osteogenesis imperfecta (OI) patients and presence of the identified variant in the parents. According to the classification system of the American College of Medical Genetics (ACMG), the discovered sequence variants were classified as pathogenic and likely pathogenic.

№	Patient ID	Variant	Variant State	Variant Type	Protein Effect	ACMG Classification	Variant in the Mother	Variant in the Father
1	VN10	c.1170+5G>C c.2197A>G#	CH *	Splice siteMissense	p.? p.(Lys733Glu)	P—PS3, PP3, PM2, PS4 LP—PM2, PM6, PM1, (PM3)	c.1170+5G>C heterozygous	-
2	VN11	c.1170+5G>C	H	Splice site	p.?	P—PS3, PP3, PM2, PS4	c.1170+5G>C heterozygous	c.1170+5G>C heterozygous
3	VN20	c.1170+5G>C	H	Splice site	p.?	P—PS3, PP3, PM2, PS4	c.1170+5G>C heterozygous	c.1170+5G>C heterozygous
4	VN33	c.1170+5G>C	H	Splice site	p.?	P—PS3, PP3, PM2, PS4	c.1170+5G>C heterozygous	c.1170+5G>C heterozygous
5	VN36-1	c.1170+5G>C	H	Splice site	p.?	P—PS3, PP3, PM2, PS4	c.1170+5G>C heterozygous	c.1170+5G>C heterozygous
6	VN36-2	c.1170+5G>C	H	Splice site	p.?	P—PS3, PP3, PM2, PS4	c.1170+5G>C heterozygous	c.1170+5G>C heterozygous
7	VN36-3	c.1170+5G>C	H	Splice site	p.?	P—PS3, PP3, PM2, PS4	c.1170+5G>C heterozygous	c.1170+5G>C heterozygous
8	VN37-1	c.1170+5G>C	H	Splice site	p.?	P—PS3, PP3, PM2, PS4	c.1170+5G>C heterozygous	c.1170+5G>C heterozygous
9	VN37-2	c.1170+5G>C	H	Splice site	p.?	P—PS3, PP3, PM2, PS4	c.1170+5G>C heterozygous	c.1170+5G>C heterozygous
10	VN42	c.1170+5G>C c.1934G>A#	CH	Splice site Missense	p.? p.(Cys645Tyr)	P—PS3, PP3, PM2, PS4 LP—PM3, PM2, PP3	c.1934G>A heterozygous	c.1170+5G>C heterozygous
11	VN43	c.1170+5G>C	H	Splice site	p.?	P—PS3, PP3, PM2, PS4	c.1170+5G>C heterozygous	c.1170+5G>C heterozygous
12	VN75	c.1224-79G>A# c.257del#	CH	Intronic Frameshift	p.(?) p.(Glu86Glyfs*22)	LP—PM3, PM2, PP3, (PS4) P—PM3, PVS1, PM2	c.257del heterozygous	c.1224-79G>A heterozygous
13	VN98	c.1170+5G>C	H	Splice site	p.?	P—PS3, PP3, PM2, PS4	c.1170+5G>C heterozygous	c.1170+5G>C heterozygous
14	VN100	c.1170+5G>C	H	Splice site	p.?	P—PS3, PP3, PM2, PS4	c.1170+5G>C heterozygous	c.1170+5G>C heterozygous
15	VN101	c.1224-79G>A#	H	Intronic	p.(?)	LP—PM3, PM2, PP3, (PS4)	NA	NA
16	VN107	c.1170+5G>C	H	Splice site	p.?	P—PS3, PP3, PM2, PS4	heterozygous	NA
17	VN114	c.1170+5G>C	H	Splice site	p.?	P—PS3, PP3, PM2, PS4	c.1170+5G>C heterozygous	c.1170+5G>C heterozygous

* Biallelic state has to be proven. # Novel variants. CH, compound heterozygous; H, homozygous; LP, likely pathogenic; NA, not available; P, pathogenic; PM1, located in a mutational hot spot and/or critical and well-established functional domain (e.g., active site of an enzyme) without benign variation; PM2, absent from controls (or at extremely low frequency if recessive) in Exome Sequencing Project, 1000 Genomes Project, or Exome Aggregation Consortium; PM3, for recessive disorders, detected in trans with a pathogenic variant; PM6, assumed de novo, but without confirmation of paternity and maternity; PP3, multiple lines of computational evidence support a deleterious effect on the gene or gene product (conservation, evolutionary, splicing impact, etc.); PS3, well-established in vitro or in vivo functional studies supportive of a damaging effect on the gene or gene product; PS4, the prevalence of the variant in affected individuals is significantly increased compared to the prevalence in controls; PVS1, null variant (nonsense, frameshift, canonical ± 1 or 2 splice sites, initiation codon, single, or multi-exon deletion) in a gene where loss of function is a known mechanism of disease.

## Data Availability

The datasets used and/or analysed during the current study are available from the corresponding author on reasonable request. The variants identified in the study were submitted to the LOVD Osteogenesis Imperfecta variant database https://databases.lovd.nl/shared/genes/ (accessed on 23 February 2022).

## Supplementary Materials

The following supporting information can be downloaded at: https://www.mdpi.com/article/10.3390/genes13030407/s1, Table S1: List of OI panel genes; Table S2: Primers used for Sanger sequencing analysis of the *P3H1* gene in patients’ siblings and parents; Table S3: Collagen type I α chain Pro986 residue 3-hydroxylation described previously in patients with *P3H1* pathogenic variants.
