# Supplementary material for: Phenotypic Variation in Vietnamese Osteogenesis Imperfecta Patients Sharing a Recessive P3H1 Pathogenic Variant
Source: Genes (Basel). 2022 Feb 24;13(3):407. doi: 10.3390/genes13030407 (PMC8950175; doi:10.3390/genes13030407)
Supplement: Supplementary file 1 [file genes-13-00407-s001.zip › genes-1583282-supplementary.pdf]

Table S1.

List of genes analysed in targeted next-generation sequencing OI and related disorders panel of 41 genes at the Genome Diagnostic Laboratory of Amsterdam UMC.

ALPL, ANO5, B3GALT6, B3GAT3, B4GALT7, BMP1, CCDC134, CHST3, COL1A1, COL1A2, CREB3L1, CRTAP, FKBP10, GORAB, IFITM5, KDELR2, LIFR, LRP5, MBTPS2, MESD, NBAS, P3H1, P4HA1, P4HB, PLOD2, PLOD3, PLS3, PPIB, SEC24D, SERPINF1, SERPINH1, SGMS2, SLC10A7, SP7, SPARC, TAPT1, TENT5A, TMEM38B, WNT1, XYLT1, XYLT2

Table S2.

Primers used for Sanger sequencing analysis of the *P3H1* gene in patients' siblings and parents.

**Exon**	**Primer**	**Sequence 5'-3'**	**Tm**	**Product (bp)**
Exon 1	Forward primer	GGTCCTGAGCATGGAACG	57.79	425
	Reverse primer	GCCAGGAGGCCACTTTC	57.51	
Intron 1	Forward primer	CGAGTGAGAAGCTAGTAGCGG	60.00	228
	Reverse primer	GCACTCTGGCATTCCGAAAAA	59.73	
Exon 2	Forward primer	TGGATGTGAGCATTATCCACC	57.79	266
	Reverse primer	ACAGAGCAAGACTCTGTCTCTAA	58.60	
Exon 5	Forward primer	TTTGACTCTTTCCTCTCAGCC	57.59	230
	Reverse primer	AGGTCTGAGTTCAGCTGG	55.81	
Exon 6	Forward primer	CAGGGCTGAGTTCTGGG	56.40	188
	Reverse primer	ATCTCTCCTGCTCACTCTTTC	56.83	
Intron 7	Forward primer	GCACTTGAGGCTTCCTGAGCCC	65.66	116
	Reverse primer	GCTCTGAGGACTGCATTCCA	59.75	
Exon 8	Forward primer	CCCTGGAATGCAGTCCT	55.56	208
	Reverse primer	CCTGGCTCCCTGACAAC	56.75	
Exon 10	Forward primer	GCTGCCCTGGCTAACATCA	60.08	179
	Reverse primer	ACCCCTTTCTCCACACTCT	57.42	
Exon 11	Forward primer	GAGCCAAACCATATCAGTCG	56.31	262
	Reverse primer	AGCACAGAGCTGAGGTACA	57.94	
Exon 12	Forward primer	AGTTCATTGGGCATTCCGTA	57.20	284
	Reverse primer	TCTGAGGAGGGCACAGAC	57.88	
Exon 13	Forward primer	AAAGGGAGGCAGGGAAG	55.44	192
	Reverse primer	CTTTGGGAACATCAGCTGTAGAA	58.67	
Exon 14	Forward primer	TCTGAGAGTGGATTACAGTCTTTG	57.97	252
	Reverse primer	GAACGGGTCACCACAGCA	59.89	
Exon 15	Forward primer	TAGCGAGTGTCTGGAGCG	58.81	257
	Reverse primer	TTCCTCTCCATGGGTCTAGT	56.75	

Table S3.

Collagen type I α chain Pro986 residue 3-hydroxylation described previously in patients with *P3H1* pathogenic variants.

Pathogenic variant	Zygosity	Protein change	Effect	3-Hyp (%)	OI severity	Reference
c.1080+1G>T	Homozygous	p.?	Splice site	0-4	II/III	(Cabral et al. 2007), (Baldridge et al. 2008)
c.1080+1G>T / g.1473+1G>T	Compound heterozygous	p.?	Splice site/ Splice site	0	II/III	(Cabral et al. 2007)
c.747delC/ c.1080+1G>T	Compound heterozygous	p.(Tyr250Metfs*87)/ p.?	Frameshift/ Splice site	10	II/III	(Cabral et al. 2007)
c.1656C>A	Homozygous	p.(Tyr552*)	Nonsense	4	III	(Cabral et al. 2007)
c.232delC	Homozygous	p.(Gln78Serfs*30)	Frameshift	15	II/III	(Baldridge et al. 2008)
c.933C>G	Homozygous	p.(Tyr311*)	Nonsense	5	III	(Baldridge et al. 2008)
c.2041C>T	Homozygous	p.(Arg681*)	Nonsense	4	II/III	(Baldridge et al. 2008)
c.1383_1389dup	Homozygous	p.(Lys464Glufs*19)	Frameshift	6	III	(Baldridge et al. 2008)
c.1170+2T>A	Homozygous	p.?	Splice site	7	II/III	(Baldridge et al. 2008)
c.628C>T	Homozygous	p.(Arg210*)	Nonsense	NA (Significantly reduced)	II/III	(Willaert et al. 2009)
c.1102C>T/ c.2055+18G>A	Compound heterozygous	p.(Arg368*)/ p.?	Nonsense /	NA (Significan	III	(Willaert et al. 2009)

			Splice site	tly reduced)		
c.2055+18G>A	Homozygous	p.?	Splice site	NA (Significantly reduced)	III	(Willaert et al. 2009)
